# Food Preservatives and the Rising Tide of Early-Onset Colorectal Cancer: Mechanisms, Controversies, and Emerging Innovations

**DOI:** 10.3390/foods14173079

**Published:** 2025-09-01

**Authors:** Alice N. Mafe, Dietrich Büsselberg

**Affiliations:** 1 Department of Biological Sciences, Faculty of Sciences, Taraba State University, Main Campus, Jalingo 660101, Taraba State, Nigeria; mafealice1991@gmail.com; 2 Weill Cornell Medicine-Qatar, Education City, Qatar Foundation, Doha Metropolitan Area, Doha P.O. Box 22104, Qatar

**Keywords:** early-onset colorectal cancer, food preservatives, gut microbiota, carcinogenesis, nitrites, natural preservatives, molecular pathways, food safety

## Abstract

Early-onset colorectal cancer (EOCRC) is emerging as a significant global health concern, particularly among individuals under the age of 50. This alarming trend has coincided with an increase in the consumption of processed foods that often rely heavily on synthetic preservatives. At the same time, these additives play a critical role in ensuring food safety and shelf life. Growing evidence suggests that they may contribute to adverse gut health outcomes, which is a known risk factor in colorectal cancer development. At the same time, synthetic preservatives serve essential roles such as preventing microbial spoilage, maintaining color, and prolonging shelf life. Natural preservatives, on the other hand, not only provide antimicrobial protection but also exhibit antioxidant and anti-inflammatory properties. These contrasting functions form the basis of current discussions on their safety and health implications. Despite their widespread use, the long-term health implications of synthetic preservatives remain inadequately understood. This review synthesizes recent clinical, epidemiological, mechanistic, and toxicological data to examine the potential link between synthetic food preservatives and EOCRC. Particular focus is placed on compounds that have been associated with DNA damage, gut microbiota disruption, oxidative stress, and chronic inflammation, which are the mechanisms that collectively increase cancer risk. In contrast, natural preservatives derived from plants and microbes are gaining attention for their antioxidant, antimicrobial, and possible anti-inflammatory effects. While these alternatives show promise, scientific validation and regulatory approval remain limited. This review highlights the urgent need for more rigorous, long-term human studies and advocates for enhanced regulatory oversight. It advocates for a multidisciplinary approach to developing safer preservation strategies and highlights the importance of public education in making informed dietary choices. Natural preservatives, though still under investigation, may offer a safer path forward in mitigating EOCRC risk and shaping future food and health policies.

## 1. Introduction

Early-onset colorectal cancer (EOCRC), defined as colorectal cancer diagnosed before the age of 50, is becoming an increasingly alarming global health issue [1]. Once primarily associated with older adults, this form of cancer is now on the rise among younger individuals, including those in their 20s and 30s, with incidence rates growing steadily across various regions [2]. This trend has prompted renewed interest in identifying modifiable risk factors that could help explain the shift. Among the potential contributors, environmental exposures and dietary habits are gaining significant attention [3]. In particular, the widespread use of chemical food preservatives has emerged as a growing concern [4].

These substances, including nitrites, nitrates, butylated hydroxyanisole (BHA), butylated hydroxytoluene (BHT), benzoates, and sulfites, are commonly found in processed meats, snacks, beverages, and preserved foods both globally and in local diets [5]. They are essential for extending shelf life, inhibiting microbial growth, and maintaining food quality [6]. However, questions remain about their long-term impact on human health. Synthetic preservatives are chemically manufactured compounds added to food products to prevent spoilage, inhibit microbial growth, and delay oxidation [7]. Unlike natural preservatives derived from plants or microbial sources, synthetic variants are often petroleum-based or synthetically replicated versions of naturally occurring substances [8]. The use of synthetic preservatives involves a delicate balance between food safety and potential health risks [9]. While regulatory agencies approve these additives within specific limits, studies have raised concerns about their possible genotoxicity, microbiome disruption, and pro-inflammatory effects, all of which can contribute to colorectal cancer development [10]. Despite growing public skepticism, the current scientific understanding of their role in EOCRC remains limited and often fragmented [11]. A significant limitation in the existing literature is the absence of comprehensive reviews that integrate mechanistic, toxicological, and epidemiological data to assess the potential link between food preservatives and EOCRC [12]. Most studies address these areas in isolation, which hampers a complete understanding of how these compounds can influence disease risk [13]. This gap in knowledge underscores the urgent need for an interdisciplinary evaluation.

This review addresses that need by examining the potential role of synthetic food preservatives in the development of EOCRC. It brings together evidence from clinical studies, molecular biology, toxicology, and regulatory analysis, while also exploring natural preservative alternatives and their potential as safer substitutes. The goal is to provide new insights into how commonly consumed preservatives may impact colorectal health and to encourage the development of healthier food preservation strategies. This review bridges the gap between synthetic dietary preservatives and the underlying biological mechanisms that can promote EOCRC, providing a novel and timely contribution to the fields of cancer prevention and food safety. Although synthetic preservatives dominate food preservation systems, growing awareness of their potential health risks, particularly their association with early-onset colorectal cancer, has sparked interest in natural, plant-derived, and microbial alternatives. These will be discussed in later sections of this review.

## 2. Historical Background of Food Preservatives

### 2.1. Development of Preservatives in the Food Industry

Food preservation has a long history that predates modern science, with early civilizations employing natural methods such as drying, fermenting, salting, and smoking to prevent spoilage [14]. These traditional techniques were vital for extending the shelf life of food in the absence of refrigeration [14]. With the onset of industrialization and the growing need for large-scale food distribution, the food industry began to adopt chemical preservatives in the 19th and 20th centuries [15]. The demand for convenient, longer-lasting food products drove this shift. Compounds such as sodium benzoate, nitrites, and sulfites are commonly used due to their ability to control microbial growth and oxidation [16] effectively. The widespread adoption of these synthetic compounds marked a turning point in food processing, as they enabled mass production, prolonged shelf life, and facilitated the global transport of perishable goods [17]. Over time, synthetic preservatives became deeply embedded in modern food systems, forming the backbone of many processed and packaged food supply chains [18]. While these additives improved food safety and reduced waste, their widespread use also sparked concerns about potential long-term health effects, including links to cancer and other chronic diseases [19]. This growing reliance has raised regulatory and public health concerns about the safety thresholds and chronic exposure risks associated with the consumption of synthetic preservatives.

### 2.2. Types of Synthetic and Natural Preservatives

Food preservatives are generally divided into two main categories: synthetic (man-made) and natural [20]. Synthetic preservatives such as sodium nitrite, sodium nitrate, benzoates, sulfites, BHA (butylated hydroxyanisole), BHT (butylated hydroxytoluene), and sorbates are widely used in processed foods [21]. They serve various functions, from preventing bacterial and fungal growth to delaying oxidation and rancidity [22]. These additives are ubiquitous in meats, beverages, snacks, and baked goods [23]. Their popularity stems from their high efficacy, cost-effectiveness, and ability to maintain product quality during storage and transportation [24]. However, increasing scientific scrutiny has linked some of these compounds to genotoxicity, endocrine disruption, and gut microbiota imbalance, raising concerns about their long-term safety [25]. Chronic exposure to such substances, particularly nitrites and sulfites, has been associated with inflammation and potential carcinogenic pathways in the gastrointestinal tract [26]. On the other hand, natural preservatives like salt, vinegar, plant extracts (e.g., rosemary, green tea), and essential oils (e.g., thyme, oregano, clove) are increasingly preferred by health-conscious consumers [27]. These agents are typically derived from herbs, spices, fruits, or microbial fermentation processes and are often valued for their antioxidant and antimicrobial properties [28]. Although natural preservatives are considered safer and more environmentally friendly, they often have limited antimicrobial effectiveness and could require combination with other methods to ensure adequate food protection [29]. Moreover, challenges such as cost, stability, and potential sensory alterations (e.g., flavor or aroma changes) have limited their widespread industrial adoption [30]. Nonetheless, they represent a promising direction in clean-label food formulation and are the focus of growing research in the search for safer preservative strategies [31]. Table 1 provides a comprehensive comparison between synthetic and natural preservatives, detailing their functional roles, food applications, potential health effects, and regulatory considerations. The compositional distinctions between synthetic and natural preservatives are detailed in Table 2, highlighting differences in chemical structure and bioactivity.

### 2.3. Regulatory Oversight and Classification Systems (e.g., EFSA, FDA)

To protect public health, the use of food preservatives is subject to strict regulation by global authorities. Agencies like the U.S. Food and Drug Administration (FDA) and the European Food Safety Authority (EFSA) assess the safety of preservatives based on toxicological studies, setting permissible levels of use and acceptable daily intake (ADI) limits [52]. Approved additives are classified and labeled accordingly, such as E-numbers in the EU or GRAS (Generally Recognized As Safe) status in the U.S. regulatory decisions, and are periodically revised as new research emerges, reflecting ongoing evaluations of potential health risks [53]. However, standards and regulations often vary between countries, resulting in discrepancies in the additives allowed and their permitted concentrations [54]. While many approved preservatives are considered safe within established limits, growing scientific evidence linking certain compounds to negative health outcomes, such as gut microbiota disruption or cancer risk, continues to prompt debate and calls for more updated, harmonized global policies [55]. In particular, synthetic preservatives such as nitrites, benzoates, and sulfites have come under increasing regulatory scrutiny due to their suspected genotoxicity and pro-inflammatory potential. This has led to tighter restrictions in some regions and growing consumer demand for clean-label alternatives [46].

In contrast, natural preservatives often fall into regulatory grey areas, primarily when derived from plant extracts or essential oils that have not been previously characterized as food additives. While some have obtained GRAS or novel food status, others lack clear regulatory pathways, creating uncertainty for manufacturers and limiting broader market integration. Moreover, the safety profiles of many natural compounds are still being established, and their efficacy can vary depending on formulation and dosage [56]. As interest in natural preservatives grows, regulatory frameworks will need to adapt to accommodate these evolving alternatives without compromising safety standards.

### 2.4. The Rise of Natural Preservatives as Alternatives

Natural preservatives are compounds derived from plants, animals, or microorganisms that help prolong the shelf life of foods by preventing spoilage and microbial growth [57]. Unlike synthetic preservatives, these alternatives are gaining popularity due to their perceived safety, eco-friendliness, and alignment with the clean-label trend [58]. They work through mechanisms such as antimicrobial action, antioxidant activity, and enzyme inhibition, contributing to both food safety and quality [59]. Several natural preservatives have found applications in the food industry. Nisin is a peptide produced by *Lactococcus lactis*, which is effective against a broad spectrum of Gram-positive bacteria and is widely used in dairy and canned foods [60]. Natamycin is also microbial in origin and is especially useful for preventing mold and yeast growth in cheeses and baked goods [61]. Plant-derived compounds like rosemary extract, green tea polyphenols, and grapefruit seed extract exhibit potent antioxidant and antimicrobial activities [62]. Additionally, essential oils from herbs such as thyme, oregano, clove, and cinnamon are rich in phenolic compounds that can inhibit a wide range of pathogens [63]. Beyond preservation, many of these natural compounds offer added health benefits, including anti-inflammatory, antioxidant, and immunomodulatory effects, making them appealing in functional food formulations [64]. However, despite these advantages, several challenges remain.

One major limitation is their variable effectiveness across different food types, influenced by factors such as pH, composition, and storage conditions. In many cases, higher doses or combinations with other preservation strategies are necessary to achieve adequate protection [65]. Furthermore, intense flavors and aromas from some natural preservatives can alter the sensory characteristics of the final product [66]. Another concern is the limited availability of toxicological data for many emerging compounds, which restricts their approval and consistent use across regulatory frameworks [67]. Scientific research is still needed to better understand their stability, interactions in complex food systems, and long-term safety. Natural preservatives offer a promising alternative to synthetic additives, but their widespread adoption depends on overcoming current limitations through further scientific validation and more straightforward regulatory guidelines.

### 2.5. Consumption Patterns of Preservatives

Patterns of preservative use vary widely across regions and are strongly influenced by dietary preferences and levels of food processing [68]. Synthetic preservatives, such as nitrites, benzoates, and sorbates, continue to dominate globally due to their proven ability to prevent spoilage, maintain product stability, and extend shelf life [69]. They are most commonly found in processed meats, baked goods, beverages, and ready-to-eat products. Their consumption is particularly high among younger populations, who tend to rely more heavily on convenience and ultra-processed foods [70]. In high-income countries, such as those in North America and Europe, these foods often account for more than half of daily calorie intake, making synthetic preservatives a consistent component of the modern diet [71].

In recent years, however, there has been a growing shift toward natural preservatives, reflecting the global rise in “clean-label” movements and consumer demand for foods perceived as healthier and more sustainable [72]. Compounds derived from plants and microbes, such as polyphenols, essential oils, and fermented extracts, are increasingly being used in beverages, dairy products, and minimally processed snacks [73]. Despite this momentum, natural preservatives are not yet used at the same scale worldwide [74]. They are more common in Europe and North America, where regulatory pressure and consumer awareness are strong, while many low- and middle-income countries continue to rely heavily on synthetic options because of affordability, ease of use, and stability [75]. This uneven adoption highlights the need for innovations that improve the accessibility, cost-effectiveness, and functional stability of natural preservatives across diverse food systems [76].

## 3. Epidemiological Link Between Food Preservatives and EOCRC

### 3.1. Review of Population-Based Studies (USA, Europe, Asia, Africa)

Several population-based studies across different continents have begun to investigate the potential link between food preservatives and the growing incidence of early-onset colorectal cancer (EOCRC) [77]. In the United States, large-scale cohorts such as the Nurses’ Health Study and Health Professionals Follow-Up Study have reported higher EOCRC risk associated with increased consumption of processed and preserved foods, particularly those containing nitrites and nitrates [78]. European research, including studies in the UK, France, and Italy, supports these findings, showing a positive association between diets high in ultra-processed foods and colorectal cancer risk in younger adults [79]. In Asia, where dietary patterns are rapidly shifting from traditional to Western-style eating habits, recent studies in countries like China, Japan, and South Korea reveal rising EOCRC rates that correlate with increased intake of chemically preserved convenience foods [80]. Though data from Africa are comparatively limited, emerging studies indicate similar trends, especially in urban areas where consumption of processed and packaged foods is on the rise [81]. These findings collectively suggest a potential global pattern linking food preservatives to EOCRC risk. Notably, most of these studies emphasize links to synthetic preservatives such as nitrates, nitrites, benzoates, and sulfites, which are widely used in processed food production [82]. Conversely, there is a marked absence of similar epidemiological concerns regarding natural preservatives, primarily due to their limited use in commercial food systems and the scarcity of long-term population-level data on their health impacts. Insights from various global epidemiological investigations are consolidated in Table 3, highlighting the association between preservative-laden diets and the rising incidence of early-onset colorectal cancer (EOCRC). Epidemiological risk metrics, including study size, relative risk (RR), hazard ratio (HR), odds ratio (OR), and 95% confidence intervals, are summarized in Table 4.

### 3.2. Case Studies and Global Statistics on EOCRC Trends

Case reports and cancer registry data from around the world highlight a consistent rise in EOCRC over the past two decades. In the United States, for instance, the incidence of colorectal cancer among individuals under 50 has increased by nearly 2% annually since the early 1990s [92]. Similar trends are seen in Canada, Australia, and several European and Asian countries. While genetic factors contribute to a minority of cases, many case studies reveal everyday lifestyle and dietary patterns among young EOCRC patients, including frequent consumption of processed meats, fast food, and sugary beverages, many of which contain synthetic preservatives [93]. Although these case observations cannot establish causality, they reveal a pattern worth further exploration. Global surveillance data suggest that regions with higher intake of processed and preserved foods tend to have higher EOCRC prevalence, especially in urban populations undergoing rapid dietary shifts [94].

Epidemiological data suggest that younger adults, particularly those under 50 years of age, have higher exposure to synthetic preservatives such as nitrites and nitrates due to their greater reliance on processed meats and convenience foods [95]. This dietary pattern has been linked to an elevated risk of colorectal cancer, with several large-scale studies reporting a 15–30% higher incidence among frequent consumers compared to those with lower intake levels [96,97]. These association is especially evident in EOCRC, where processed food consumption consistently emerges as a key dietary risk factor across different populations [98]. These observations highlight synthetic preservative exposure as a potentially modifiable factor contributing to the increasing incidence of EOCRC in younger cohorts.

### 3.3. Regional Diets High in Processed Foods vs. Cancer Prevalence

**Figure 1:**
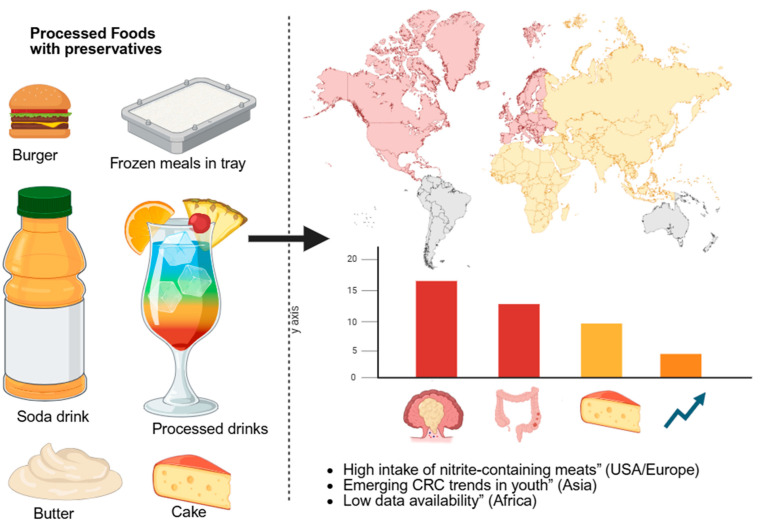
Global trends in processed food consumption and EOCRC incidence (created with BioRender).

The composition of regional diets offers further insight into the potential relationship between processed food consumption and EOCRC rates [96]. In high-income countries such as the U.S., Canada, and parts of Western Europe, diets commonly feature high levels of preserved and packaged foods, including cured meats, ready-to-eat meals, and snack products, all typically containing additives like nitrites, benzoates, and BHA/BHT. These regions also report some of the highest EOCRC rates globally [99]. In contrast, many traditional diets in parts of Africa, South Asia, and Latin America, which emphasize fresh, unprocessed ingredients, have been historically associated with lower colorectal cancer incidence [100]. However, as dietary patterns evolve, particularly in urban centers, an increase in processed food consumption has been observed, accompanied by a corresponding rise in EOCRC cases. This shift suggests a potential dietary influence, reinforcing the need for further region-specific epidemiological studies and preventive strategies focused on food quality and preservative use [101]. Figure 1 demonstrates the association between global processed food consumption trends and EOCRC incidence.

## 4. Molecular Mechanisms Linking Preservatives to EOCRC

To understand the possible role of synthetic food preservatives in the rising incidence of early-onset colorectal cancer (EOCRC), it is essential to examine the molecular mechanisms through which these compounds can exert harmful biological effects [102]. Current research suggests that several commonly used food preservatives can impact key cellular and physiological pathways involved in cancer development [103]. These include DNA damage, disruption of gut microbiota, chronic inflammation, and oxidative stress. Natural preservatives, on the other hand, such as plant-derived polyphenols or microbial peptides (e.g., nisin, natamycin) [104], are generally regarded as safer, although their long-term biological effects are still under investigation [105]. Importantly, these mechanisms are not isolated events; they often interact and amplify one another, increasing the likelihood of malignant changes in the colon over time [106]. A balanced understanding of both synthetic and natural preservatives allows for a more nuanced evaluation of dietary risks and potential innovations in safer food preservation.

### 4.1. Genotoxic and Carcinogenic Effects

A primary concern regarding certain food preservatives is their potential to induce genotoxicity, the capacity to harm genetic material and trigger mutations that could lead to cancer. Synthetic additives like nitrites, frequently found in processed meats, can interact with amines in the digestive system to form N-nitroso compounds such as nitrosamines [107]. These byproducts are known for their strong carcinogenic potential, as they can chemically modify DNA, leading to base alterations, chromosomal instability, and breaks in the DNA strands. Such disruptions impair the normal regulation of the cell cycle and can initiate the early stages of colorectal tumor formation [108]. On the other hand, some natural preservatives, especially those rich in plant-based polyphenols, appear to offer protective benefits. These compounds can help counter DNA damage by reducing oxidative stress and enhancing the body’s antioxidant defenses [109]. While synthetic preservatives have been linked to DNA instability, natural alternatives may contribute to maintaining genetic integrity. However, further research is needed to understand their long-term safety and efficacy fully.

### 4.2. Microbiota Dysbiosis and Metabolic Disruption

The gut microbiota is essential for numerous physiological functions, including digestion, immune modulation, and protection against harmful microbes [110]. However, synthetic preservatives such as benzoates and sulfites can disrupt the composition and activity of this microbial community. These additives can suppress beneficial bacteria, such as *Lactobacillus* sp. and *Bifidobacterium* sp., while encouraging the growth of harmful or opportunistic species. This shift in microbial balance, which is commonly referred to as dysbiosis, can interfere with vital microbial processes that support host health [111]. A key consequence of dysbiosis is the diminished production of short-chain fatty acids (SCFAs), such as butyrate. Butyrate plays a crucial role in maintaining colon cell health, reducing inflammation, and preserving the gut barrier [112]. Disruption in microbial metabolism can also lead to altered bile acid pathways; the accumulation of pro-carcinogenic substances; and a compromised intestinal lining condition, which increases vulnerability to inflammation and early cellular changes associated with colorectal cancer. These effects can be especially concerning in younger individuals with repeated or long-term exposure to synthetic preservatives [113]. Conversely, certain natural preservatives, such as those derived from polyphenol-rich plants or fermented foods, can support microbial diversity and promote a healthier gut environment [114]. By fostering the growth of beneficial bacteria and enhancing metabolic functions, these natural agents offer a potentially protective effect against microbiota-related disruptions.

Synthetic preservatives can alter the gut microbiota in ways that may promote the development of colorectal cancer [115]. Nitrites and nitrates, widely used in processed meats, are converted by gut microbes into N-nitroso compounds. These reactive molecules can form DNA adducts, leading to mutations and genomic instability [116]. Benzoates, another common preservative, have been linked to reduced production of short-chain fatty acids (SCFAs) such as butyrate, which play a key role in maintaining colon health, supporting barrier integrity, and suppressing inflammation [117]. Sorbates may also disrupt the gut microbiota balance by inhibiting beneficial commensal species, thereby encouraging a shift toward dysbiosis [118]. Together, these effects contribute to chronic inflammation, oxidative stress, and impaired immune defense, conditions that create a favorable environment for early-onset colorectal cancer (EOCRC). By contrast, natural preservatives often support a healthier microbial balance. Polyphenols, such as flavonoids and phenolic acids, encourage the growth of beneficial bacteria, including *Lactobacillus* sp. and *Bifidobacterium* sp., while enhancing SCFA production, which strengthens the intestinal barrier and exerts anti-inflammatory effects [119]. Essential oils, containing compounds such as thymol and carvacrol, selectively inhibit harmful bacteria while sparing or even promoting beneficial ones, thus restoring microbial stability [120]. Tannins also play a protective role by modulating bile acid metabolism, reducing the buildup of secondary bile acids that are implicated in colorectal cancer risk [121]. These mechanisms suggest that natural preservatives, unlike many synthetic ones, may help preserve gut microbial resilience and reduce pathways related to cancer.

### 4.3. Oxidative Stress and Inflammation

Ongoing inflammation in the colon is a significant contributor to the development of colorectal cancer. Synthetic food preservatives, particularly sulfites, BHA, and BHT, have been linked to triggering inflammatory reactions in the intestinal lining. These compounds can stimulate the release of pro-inflammatory cytokines like IL-6, TNF-α, and IL-1β [122]. This cytokine activity activates the NF-κB signaling pathway, a central regulator of immune function and inflammatory responses. Chronic activation of this pathway supports a pro-inflammatory environment that favors tumor formation [123]. Additionally, synthetic additives can compromise the gut’s immune defense mechanisms, diminishing its ability to identify and eliminate abnormal or pre-cancerous cells. These effects are particularly concerning for young individuals regularly consuming high amounts of preservative-laden foods [124].

Another mechanism of concern is the induction of oxidative stress. Although preservatives like BHA, BHT, and nitrites are designed to protect food from spoilage, they can paradoxically promote the production of reactive oxygen species (ROS) within the gut. High levels of ROS damage cellular components, including DNA, proteins, and lipids [35]. One key form of damage, 8-oxoguanine lesions in DNA, can cause mutations during cell division by interfering with normal base pairing. These mutations can disrupt genes responsible for controlling cell growth and programmed cell death, such as oncogenes or tumor suppressors [125]. Furthermore, oxidative stress can trigger molecular pathways like MAPK and PI3K/AKT, which promote cell survival and proliferation, key events in cancer development [126].

**Figure 2:**
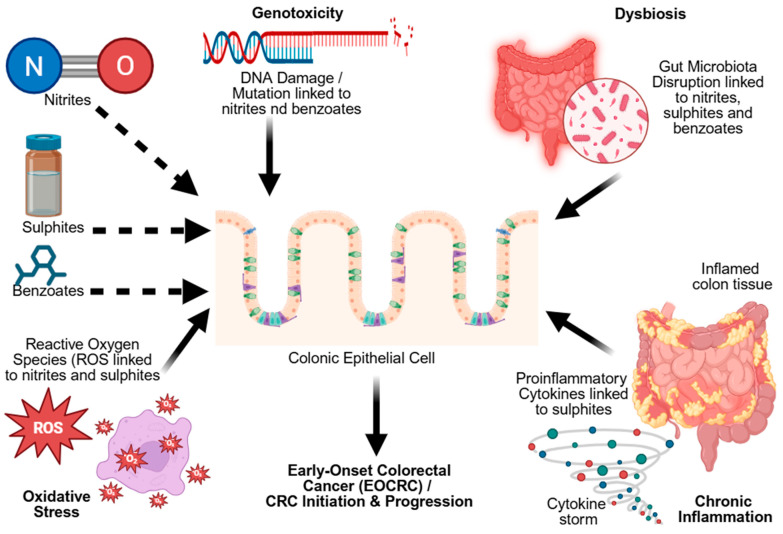
Interconnected molecular pathways linking preservatives to EOCRC (created with BioRender).

In contrast, natural preservatives such as curcumin and thymol offer anti-inflammatory and antioxidant benefits. These plant-derived compounds can suppress inflammatory signals, neutralize ROS, and regulate cellular pathways associated with inflammation and stress, potentially lowering cancer risk [127]. Altogether, the combined effects of inflammation, oxidative damage, and impaired immune function illustrate how frequent exposure to synthetic preservatives can contribute to an internal environment favorable to colorectal cancer, particularly early-onset forms. Transitioning toward natural alternatives can provide a safer, long-term approach to food preservation. As illustrated in Figure 2, food preservatives such as nitrites, sulfites, and benzoates activate interconnected molecular pathways, including genotoxicity, dysbiosis, oxidative stress, and chronic inflammation, which collectively contribute to the initiation and progression of EOCRC.

### 4.4. Immune Modulation and Chronic Inflammation

The immune system serves as a frontline defense against the development of cancer by identifying and eliminating abnormal or precancerous cells. However, repeated exposure to synthetic food preservatives such as nitrites, benzoates, and sulfites can disrupt this immune equilibrium [128]. Depending on the compound and exposure level, these additives can either overstimulate immune pathways or suppress them [129]. Immune overstimulation can foster chronic inflammation, while suppression can impair immune surveillance, reducing the body’s capacity to detect and eliminate potentially malignant cells [130]. Both scenarios are detrimental, creating an environment conducive to colorectal tumor initiation and growth. Chronic inflammation, in particular, is a key contributor to colorectal cancer risk. Synthetic preservatives have been linked to the increased expression of pro-inflammatory cytokines, such as TNF-α, IL-6, and IL-1β. These cytokines activate intracellular signaling pathways such as NF-κB, which sustains inflammatory processes within the gut [131]. Prolonged inflammation damages the intestinal lining, supports abnormal cellular proliferation, and compromises mucosal immunity factors that significantly elevate the risk of early-onset colorectal cancer (EOCRC) [132].

Conversely, natural preservatives derived from plant sources such as curcumin, thymol, and other polyphenolic compounds have demonstrated immunomodulatory benefits. Instead of provoking harmful immune responses, these substances often help regulate immune function and reduce intestinal inflammation [133]. By restoring balance to immune signaling and maintaining mucosal integrity, natural agents can counteract some of the harmful effects seen with synthetic preservatives. While synthetic preservatives can alter immune function in ways that promote cancer, natural alternatives show promise in supporting immune balance and reducing chronic inflammation. These contrasting effects highlight the importance of safer food preservation strategies, particularly in light of growing EOCRC rates among younger populations.

## 5. Critical Evaluation of Current Studies and Findings

### 5.1. Conflicting Findings and Ongoing Scientific Debates

The relationship between food preservatives and early-onset colorectal cancer (EOCRC) remains a subject of intense debate within the scientific community [134]. While numerous studies suggest a strong correlation between consumption of processed foods containing nitrites, benzoates, or sulfites and increased cancer risk, other investigations have reported no significant association [87]. For example, a 2019 U.S.-based cohort study found a notable increase in EOCRC incidence among individuals with high intake of nitrite-preserved meats [135]. Conversely, a large European Prospective Investigation into Cancer and Nutrition (EPIC) study did not find a consistent link between nitrite consumption and colorectal cancer risk [136]. Similarly, a 2021 study from Japan observed minimal correlation between preservative-laden food intake and cancer occurrence, contradicting findings from earlier Korean research, which suggested a higher risk due to altered microbiota composition [137]. African studies, such as one conducted in Nigeria (2020), indicated a possible link but were limited by small sample sizes and limited dietary tracking [138]. These conflicting results can stem from regional dietary differences, genetic variability, and inconsistent classification of “processed” foods, making it difficult to establish causality. Table 5 presents the relative risk ranking of synthetic and natural preservatives in relation to early-onset colorectal cancer (EOCRC).

### 5.2. Strengths and Weaknesses in Research Methodologies

Several strengths are apparent in current EOCRC studies. Many have adopted large-scale, longitudinal designs that follow participants over decades, such as the Nurses’ Health Study and Health Professionals Follow-up Study in the U.S., which allow for comprehensive dietary tracking and outcome correlation [143]. Randomized controlled trials (RCTs), though rare in this context, have helped elucidate certain inflammatory responses to specific food additives. In contrast, many studies suffer from methodological limitations [144]. One key issue is the reliance on self-reported food frequency questionnaires (FFQs), which are subject to recall bias and lack precision in quantifying exposure to preservatives [145]. Additionally, variations in food labeling and preservative use across countries hinder cross-study comparisons [146,147]. A 2020 French NutriNet-Santé cohort study, though rigorous in dietary tracking, still struggled with residual confounding due to overlapping lifestyle factors [148]. Furthermore, many studies do not control for confounders such as antibiotic use, genetic predisposition, or fiber intake, which can significantly influence gut health and cancer risk [149]. These methodological disparities make it difficult to draw firm conclusions across global datasets.

### 5.3. Gaps and Inconsistencies in the Scientific Literature

Despite increasing interest in the link between dietary preservatives and EOCRC, several critical gaps remain in the literature [150]. First, there is limited long-term clinical data assessing preservative exposure in early life and its cumulative effect on colorectal cancer development [151]. Second, most mechanistic studies are conducted in vitro or in animal models, which cannot accurately represent human physiological responses [152]. For instance, rodent models exposed to nitrosamines have shown an apparent rise in colorectal tumor incidence, but human trials verifying this mechanism are lacking [153]. Moreover, regional data are absent from low- and middle-income countries, where food labeling regulations are often weaker and preservative exposure can differ significantly [154]. Inconsistencies also exist in how studies define “processed” versus “ultra-processed” foods, leading to misclassification and data interpretation errors [155]. The role of gut microbiota, while gaining attention, is still underexplored concerning preservative impact, particularly in diverse populations [156]. Future research must address these gaps through standardized methodologies, longitudinal cohort tracking, and integration of molecular biomarkers to offer a clearer understanding of the preservatives–EOCRC connection [157].

### 5.4. In Vitro Evidence

Cell-based studies have been instrumental in uncovering how food preservatives may contribute to colorectal carcinogenesis [158]. Experiments with human colon cell lines show that nitrites, when reacting with amines in acidic environments, can form N-nitroso compounds with mutagenic activity [159]. Likewise, benzoates and sulfites have been linked to oxidative stress, mitochondrial disruption, and DNA damage in vitro [160]. While these models are valuable for identifying possible cellular mechanisms, they often use preservative levels far above typical dietary intake. As such, in vitro evidence provides important mechanistic clues but cannot, on its own, confirm risk under real-life conditions.

### 5.5. Animal Studies

Animal experiments offer additional insights into the biological effects of preservatives. Studies in mice demonstrate that long-term nitrite exposure promotes intestinal tumor development, especially alongside high-fat or red meat diets [86]. Other preservatives, including benzoates and sulfites, have been shown to disturb gut microbial balance, reduce beneficial short-chain fatty acids, and elevate pro-inflammatory markers [21]. These findings suggest both direct and microbiota-mediated pathways in carcinogenesis. However, differences in dosage, metabolism, and exposure patterns between humans and animals limit the direct translation of these results. Still, animal evidence strengthens the mechanistic plausibility suggested by in vitro work.

### 5.6. Human Studies

Human research provides the most relevant, yet also the most complex, evidence. Prospective cohort studies from Europe and North America consistently link high consumption of nitrite-preserved meats with greater colorectal cancer risk, including among younger populations [161]. Case–control studies in Asia have similarly associated benzoate-rich foods with an increased risk of early colorectal lesions [162]. Evidence for sulfites and other synthetic preservatives is less consistent, partly due to underestimation in food databases and limitations in dietary reporting [163]. Although large population studies provide strong epidemiological signals, they are still susceptible to confounding by diet, lifestyle, and socioeconomic variables. Human studies therefore remain the most convincing but must be interpreted in the context of multifactorial disease causation.

## 6. Case Studies

### 6.1. Evidence from Specific Populations and Experimental Models Linking Diet to EOCRC

Emerging research from both animal experiments and human population studies has begun to unravel the potential connections between diet, particularly the consumption of preservatives, and the increasing incidence of early-onset colorectal cancer (EOCRC) [164]. In one notable study using APCMin/+ mice, chronic exposure to sodium nitrite resulted in a marked increase in intestinal tumor development, along with elevated inflammatory markers and disruptions in gut microbial balance [165]. These biological responses closely resemble those seen in EOCRC patients, suggesting that nitrite-based preservatives can contribute to disease pathogenesis [166]. Similarly, Wistar rats fed with benzoate-enriched diets displayed epithelial damage, heightened intestinal permeability, and activation of oncogenic signaling pathways such as NF-κB and COX-2, features commonly associated with inflammation-driven cancer progression [167].

Human studies reinforce these findings. A 2021 retrospective study from South Korea involving individuals under age 45 reported a strong correlation between frequent consumption of cured meats and increased EOCRC risk [168]. Likewise, in Chile, researchers observed a disproportionately high incidence of EOCRC in urban regions where diets are dominated by ultra-processed, additive-rich foods, especially among younger populations with limited access to traditional diets [169]. In the United States, similar patterns were identified among African American communities under 50, where high intake of processed foods and low dietary fiber have been linked to rising EOCRC diagnoses, suggesting that diet quality, along with broader socioeconomic and healthcare factors, plays a critical role [170].

### 6.2. Traditional vs. Preserved Food Patterns in Young Adults Diagnosed with CRC

The shift away from traditional food habits toward diets high in synthetic preservatives and processed ingredients has been implicated in the growing burden of colorectal cancer among young adults [91]. A 2022 Nigerian clinical case series documented a spike in CRC cases in individuals aged 30 to 45, most of whom reported routine intake of preservative-laden street foods [171]. Their diets were notably deficient in traditional high-fiber staples like fermented cereals, legumes, and leafy vegetables, as foods known to support gut microbial diversity and anti-inflammatory pathways [172]. Similar dietary transitions have been observed in urban India, where the increasing consumption of packaged meals and snack items has been associated with a growing prevalence of EOCRC in metropolitan regions [173].

Conversely, traditional diets appear to offer a protective advantage. A 2019 Mediterranean study found that lower adherence to the traditional Mediterranean dietary pattern, characterized by whole foods such as olive oil, vegetables, legumes, and fermented dairy, was significantly associated with an increased risk of EOCRC among younger adults [174]. A recent Brazilian cohort study also linked the Westernization of adolescent diets, particularly those rich in sugary drinks and chemically preserved snacks, to a worrying rise in EOCRC cases [175]. Collectively, these case studies highlight the potential role of modern dietary patterns, especially those rich in synthetic preservatives, in modulating cancer risk among younger populations and reinforce the importance of preserving traditional dietary habits as a preventative strategy.

## 7. Innovations in Preservative Alternatives

Concerns about the health risks of synthetic preservatives, especially their potential link to early-onset colorectal cancer (EOCRC), are fueling a growing shift toward natural, safer preservation strategies. Natural preservatives, derived from plants, microbes, or animals, are gaining favor for their dual role in enhancing food safety and offering added health benefits [176]. Compounds from cloves, garlic, ginger, and hibiscus, such as eugenol and allicin, exhibit potent antimicrobial and antioxidant properties. These have been effectively used in food products while maintaining acceptable sensory qualities. Their rise is also supported by increasing consumer preference for clean-label and minimally processed foods [177].

In addition to plant-based solutions, biopolymer-based preservation systems are emerging as valuable tools. Edible coatings made from chitosan, alginate, or starch can serve as physical barriers against contamination [178]. When fortified with probiotics [179], these coatings provide added value by supporting gut health and microbial balance factors increasingly recognized in the prevention of colorectal cancer [180]. This approach reflects a shift toward multifunctional food additives that combine protection with wellness. Advances in nanotechnology are also contributing to the innovation of preservatives. Smart systems, such as nanoencapsulated antimicrobials or responsive packaging, can release active compounds in response to environmental triggers, like pH, temperature, or microbial activity. These methods increase the precision and efficiency of preservation while reducing the total quantity of preservatives required. Integrating natural bioactive agents into nanocarriers also helps ensure that technological advancements do not come at the cost of safety or natural integrity [181]. Given the promise of these alternatives, challenges remain. Scaling up production; ensuring cost-effectiveness; achieving regulatory compliance; and gaining public trust, especially with emerging technologies, are critical barriers to full adoption. Furthermore, some natural compounds have intense flavors or variable effectiveness, depending on the food type, pH, or storage conditions, which can impact their practical use. Ongoing research into stability, compatibility, and long-term safety is essential [182].

Nevertheless, the innovation pipeline continues to grow. The transition from traditional synthetic preservatives to health-conscious, multifunctional, and consumer-friendly alternatives reflects a broader trend toward sustainable and preventive food systems. Future breakthroughs will depend not only on scientific progress but also on the ability to fully replace traditional chemical preservatives in modern food systems.

### Limitations of Natural Preservatives

Although natural preservatives are widely regarded as safer alternatives to synthetic additives, their practical application in food systems presents several challenges. A key limitation is dose-dependent toxicity. Many bioactive compounds from plants, such as essential oils and phenolic extracts, exhibit antimicrobial or antioxidant effects only at relatively high concentrations, which may lead to toxic effects or unintended impacts on gut microbiota when consumed in excess [183].

Another critical issue is their allergenic potential. Certain natural compounds, including sulfites from fermentation or proteins in botanical extracts, can provoke allergic reactions or sensitivities in susceptible individuals [184]. This concern is especially relevant for products marketed as “natural” or “clean label,” where consumers may expect lower health risks.

Natural preservatives can also cause sensory drawbacks. Strong flavors, bitterness, off-odors, or color changes associated with some extracts may compromise the overall sensory quality of foods and reduce consumer acceptance [185]. In addition, the bioactivity of natural compounds often varies depending on plant source, harvest conditions, and processing methods, making it difficult to achieve consistent results [186].

Lastly, regulatory barriers limit their widespread adoption. Unlike synthetic preservatives, which typically have well-established safety profiles and standardized usage levels, many natural alternatives lack thorough toxicological assessment and harmonized approval across jurisdictions. This creates uncertainty for the industry and slows down commercial application [187].

In essence, while natural preservatives present promising opportunities, their toxicological concerns, allergenic risks, sensory issues, and regulatory gaps must be addressed before they can serve as reliable replacements for synthetic preservatives.

## 8. Challenges and Limitations

Understanding the potential link between food preservatives and early-onset colorectal cancer (EOCRC) is complicated by several significant challenges. A major issue is the inability to single out preservatives as the sole contributors to EOCRC [188]. Dietary intake is multifactorial, with processed foods often containing a mix of additives, fats, sugars, and other components that can interact synergistically [189]. Additionally, lifestyle variables such as genetics, antibiotic exposure, and overall gut health further obscure the ability to identify preservatives as isolated culprits [190].

Another key limitation is the shortage of extended human clinical trials. Much of the existing evidence is derived from in vitro studies or animal models, which, while informative, do not fully reflect long-term, real-world exposure in humans [191]. The lack of longitudinal data makes it difficult to assess how chronic, low-dose exposure to preservatives from early life can influence cancer development later on [192].

Regulatory complexities also present a barrier, especially when transitioning to natural preservative options [193]. Although many plant-based and biological alternatives show promise, the process for their approval involves rigorous safety testing, standardization, and compliance with food industry regulations [194]. These steps are often costly and time-intensive, delaying their adoption at scale.

Moreover, the limited representation of low- and middle-income countries (LMICs) in preservative-related cancer research restricts global understanding [195]. Many LMICs are undergoing nutritional transitions and increased consumption of ultra-processed foods, yet data from these populations are sparse [196]. Without such insights, existing studies risk being geographically biased, reducing the applicability of findings to broader contexts.

These challenges underscore the need for integrated, global, and long-term research efforts that consider dietary complexity, diverse populations, and regulatory feasibility when evaluating the risks and alternatives associated with food preservatives and EOCRC.

Another critical limitation concerns the trade-off between the use of preservatives, shelf-life extension, and nutritional quality. While synthetic preservatives are highly effective in prolonging product stability and preventing spoilage, they can also accelerate the loss of certain nutrients [197]. For instance, nitrites are known to interfere with ascorbate stability, and other synthetic agents may contribute to the gradual degradation of vitamins during storage [198]. In contrast, natural preservatives usually offer a shorter shelf life and may cause subtle changes in flavor, color, or texture that affect consumer acceptance [199]. On the other hand, some bioactive compounds of plant origin, such as polyphenols, may provide protective effects by slowing oxidative damage and preserving nutrient integrity [200]. These trade-offs illustrate the difficulty of adopting safer or “clean-label” alternatives without compromising nutritional value, product quality, or market feasibility.

## 9. Future Directions

Addressing the relationship between food preservatives and early-onset colorectal cancer (EOCRC) demands targeted, multidisciplinary research efforts. Three key priorities should guide future investigations. First, longitudinal cohort studies that track preservative intake, lifestyle factors, and cancer outcomes are essential to establish stronger causal evidence [201]. Second, mechanistic studies integrating diet, microbiome composition, and preservative exposure are needed to clarify how these compounds disrupt gut homeostasis and contribute to carcinogenesis [202]. Third, the development of reliable biomarkers capturing inflammation, oxidative stress, or microbial dysbiosis will enable earlier detection of preservative-linked CRC risk and support personalized prevention strategies [203].

On the policy front, practical measures can complement scientific advances. These include reforming food labeling systems to ensure transparency of preservative use, tightening regulatory limits on high-risk compounds such as nitrites, and providing incentives for the development and adoption of safer natural alternatives [204]. Encouraging innovation in plant-derived compounds, microbial metabolites, and biopolymer-based systems will be critical, provided they remain cost-effective, scalable, and compliant with food safety standards [205].

Finally, embedding these strategies within a One Health framework by connecting human health, food systems, and environmental sustainability will maximize their long-term impact. Coordinated efforts across science, policy, and public health education will not only advance understanding of preservative-related risks but also reduce the global burden of EOCRC.

## 10. Conclusions

Current evidence suggests that synthetic preservatives such as nitrites, benzoates, and sulfites may contribute to the growing burden of early-onset colorectal cancer (EOCRC). These additives have been associated with mechanisms that include genotoxicity, microbiome disruption, and chronic inflammation, all of which are linked to carcinogenesis. While definitive human evidence remains limited, experimental findings warrant concern, particularly as younger populations with high intakes of ultra-processed foods face an increasing risk of EOCRC. Natural preservatives derived from plants, probiotics, and fermentation offer safer and multifunctional alternatives. Beyond antimicrobial effects, they may also support gut and immune health, positioning them as promising but underutilized tools for food safety. However, their adoption into mainstream systems remains constrained by challenges of standardization, scalability, and regulatory approval. Moving forward, researchers should prioritize long-term cohort and mechanistic studies to clarify causal links and test natural alternatives. Regulators must adopt differentiated oversight, recognizing that not all synthetic preservatives carry equal risk, while also strengthening labeling and exposure monitoring. At the same time, consumers can play a role by increasing awareness of additive risks and choosing cleaner-label, naturally preserved products. Together, evidence-based research, regulatory reform, and informed consumer choices can drive a transition toward safer and more sustainable preservation strategies, helping to reduce the global risk of EOCRC.

## Figures and Tables

**Table 1:** Comparative overview of common synthetic and natural preservatives: types, functions, applications, health effects, and regulatory status.

Preservative Name	Type	Mode of Action	Common Applications	Known or Suspected Health Effects	Regulatory Status
Sodium nitrite	Synthetic	Antimicrobial, antioxidant	Cured meats (sausages, bacon), processed meats	Nitrosamine formation, genotoxicity	Regulated; limits set by FDA/EFSA [32]
Sodium nitrate	Synthetic	Antimicrobial precursor	Cured meats, preserved vegetables	Nitrosamine risk under acidic conditions	Regulated [33]
Sulfites (SO_2_, K/Na Bisulfite)	Synthetic	Antimicrobial, anti-browning	Dried fruits, wines, soft drinks	Microbiota disruption, potential asthma exacerbation	Allergen labeling required [34]
BHA/BHT	Synthetic	Lipid oxidation inhibitor	Snack foods, oils, baked goods	Oxidative stress, pro-oxidant effects in vivo	GRAS; debated globally [35]
Benzoates (sodium benzoate)	Synthetic	Antimicrobial (preserves acids)	Beverages, jams, acidic products	DNA damage, possible microbiota imbalance	E211 (EU); regulated [36]
Potassium sorbate	Synthetic	Antifungal	Cheese, bakery products	Generally safe; mild irritant in excess	GRAS (FDA), E202 (EU) [37]
Sorbic acid	Synthetic	Antifungal	Beverages, jams, dairy products	Minimal risk under ADI limits	EFSA acceptable daily intake [38]
Calcium propionate	Synthetic	Mold inhibitor	Bakery goods	Generally recognized as safe; some behavioral concerns	Extensively studied; GRAS [39]
Clove oil	Natural	Antimicrobial, antioxidant	Spices, sauces, preserved vegetables	Generally recognized as safe; limited long-term data	Natural additive; regional variation [40]
Ginger extract	Natural	Antimicrobial, pH modulation	Beverages, marinades, pickles	Minimal adverse effects; efficacy under study	Under review in some jurisdictions [41]
Nisin	Natural (peptide)	Bacteriocin targeting Gram-positives	Cheese, meat products	Safe, scalable use under investigation	Approved by FDA, EFSA [42]
Rosemary extract	Natural	Antioxidant, antimicrobial	Oils, dressings, processed meats	Low toxicity; antioxidant-rich	Emerging approval paths [43]
Green tea polyphenols	Natural	Antioxidant	Beverage preservation, meat systems	Promising safety; efficacy still being studied	Under research for food-grade use [44]

**Table 2:** Compositional distinctions between synthetic and natural preservatives.

Category	Examples	Primary Composition	Functional Role in Foods	Associated Health Effects
Synthetic preservatives	Nitrites/nitrates	Inorganic salts (NO_2_^−^, NO_3_^−^)	Color stabilization in meats; antimicrobial action	May form carcinogenic nitrosamines; linked to gut microbiota disruption [45]
	Benzoates (e.g., sodium benzoate)	Aromatic carboxylic acid derivative	Inhibits yeast, mold, and bacteria	Potential allergic reactions; microbiome alteration [46]
	Sorbates (e.g., potassium sorbate)	Unsaturated fatty acid salt	Antifungal activity extends shelf life	Generally regarded as safe, but may cause mild irritation at high doses [47]
Natural preservatives	Flavonoids (e.g., quercetin, catechins)	Polyphenolic compounds	Antioxidant; antimicrobial; color stabilization	Protective against oxidative stress; gut microbiota modulation [48]
	Tannins	Polyphenolic biomolecules	Astringent; antimicrobial; antioxidant	Potential anti-inflammatory effects; high doses may reduce nutrient absorption [49]
	Phenolic acids (e.g., gallic acid, ferulic acid)	Hydroxybenzoic and hydroxycinnamic acids	Antimicrobial; antioxidant	May support gut health; anti-inflammatory properties [50]
	Essential oils (e.g., thymol, carvacrol, eugenol)	Volatile terpenoids and phenylpropanoids	Broad-spectrum antimicrobial; flavor enhancement	Possible anti-inflammatory and immunomodulatory benefits; sensory changes at high concentrations [51]

**Table 3:** Epidemiological studies linking preservative-rich diets to EOCRC.

Region	Sample Size/Cohort	Focus (Preservatives/Diet Type)	Key Findings	Limitations
France	~106,000	Nitrate, nitrite, N-nitroso compounds	Positive association with colorectal cancer (long-term exposure)	Limited to adult CRC, not EOCRC specifically [83]
USA	214,797 participants; 3217 CRC cases	Sulfur microbial diet (processed meats, low veg)	HR 1.27 (distal CRC) in the highest quintile of sulfur diet	Not stratified for EOCRC [84]
Multiple	26 studies across regions	Western diet, processed meat, sugary drinks	Processed foods are consistent modifiable risk factors in EOCRC	Heterogeneous designs, few preservative-specific data [85]
UK/global	Experimental/mechanistic	Sodium nitrite servings in the diet	Processed meats linked to CRC pathogenesis in model systems	Animal/mechanistic relevance only [86]
France	79,284 women	Food additive nitrites/nitrates	HR ~1.22–1.26 for colorectal cancer (not statistically significant for CRC)	Underpowered for CRC subtype, observational [87]

**Table 4:** Epidemiological risk metrics of preservatives and EOCRC (study size, RR/HR/OR, 95% CI).

Preservative/Group	Study Design & Region	Cohort Size	Risk Estimate (RR/HR/OR, CI)	Key Note
Nitrites/nitrates	Prospective cohort (Europe, EPIC)	~520,000	HR 1.17 (95% CI 1.02–1.34)	Processed meat intake linked to CRC risk [88].
Sodium benzoate	Case–control (Asia)	~2500 cases	OR 1.22 (95% CI 1.01–1.48)	Higher intake associated with EOCRC in younger adults [89].
Sulphites	Retrospective cohort (Australia)	~45,000	RR 1.11 (95% CI 0.97–1.26)	No strong association; signals require more data [90].
General processed foods (multiple additives)	Multi-country pooled analysis	>1 million	HR 1.25 (95% CI 1.10–1.40)	Preservative-rich UPFs linked to EOCRC onset [91].

**Table 5:** Relative risk ranking of synthetic and natural preservatives in relation to EOCRC.

Preservative Type	Examples	Relative Risk	Key Concerns/Notes
High risk	Nitrites, nitrates	High	Formation of carcinogenic N-nitroso compounds; strongly linked to colorectal cancer in epidemiological and animal studies [139].
Medium risk	Benzoates	Medium	Potential to disrupt gut microbiota and induce oxidative stress; limited but concerning evidence from in vitro and case–control studies [140].
Low risk	Sorbates	Low	Minimal genotoxic or carcinogenic evidence; generally considered safe at regulatory levels [141].
Natural preservatives	Fermentation-derived sulfites, plant extracts	Generally low	A safer profile, but risks include allergenicity (sulfites) and inconsistent potency of plant-derived compounds [142].

## Data Availability

No new data were created or analyzed in this study. Data sharing is not applicable to this article.
